# Revealing the expression profile of genes that encode the Subcortical Maternal Complex in human reproductive failures

**DOI:** 10.1590/1678-4685-GMB-2023-0141

**Published:** 2023-12-11

**Authors:** Marília Körbes Rockenbach, Lucas Rosa Fraga, Thayne Woycinck Kowalski, Maria Teresa Vieira Sanseverino

**Affiliations:** 1 Universidade Federal do Rio Grande do Sul (UFRGS), Programa de Pós-Graduação em Genética e Biologia Molecular, Porto Alegre, RS, Brazil.; 2 Universidade Federal do Rio Grande do Sul (UFRGS), Instituto de Ciências da Saúde, Departamento de Ciências Morfológicas, Porto Alegre, RS, Brazil.; 3 Universidade Federal do Rio Grande do Sul (UFRGS), Programa de Pós-Graduação em Medicina: Ciências Médicas, Porto Alegre, RS, Brazil.; 4 Hospital de Clínicas de Porto Alegre, Centro de Pesquisa Experimental, Laboratório de Medicina Genômica, Porto Alegre, RSBrazil.; 5 Hospital de Clínicas de Porto Alegre, Núcleo de Bioinformática, Porto Alegre, RS, Brazil.; 6 Hospital de Clínicas de Porto Alegre, Serviço de Genética Médica, Porto Alegre, RS, Brazil.; 7 Centro Universitário CESUCA, Cachoeirinha, RS, Brazil.; 8 Pontifícia Universidade Católica do Rio Grande do Sul, Porto Alegre, RS, Brazil.

**Keywords:** Decidua, chorionic villus, maternal effect genes, transcriptome, Pearson’s correlation

## Abstract

The Subcortical Maternal Complex (SCMC) is composed of maternally encoded proteins required for the early stages of embryo development. Here we aimed to investigate the expression profile of the genes that encode the individual members of the SCMC in human reproductive failures. To accomplish that, we selected three datasets in the Gene Expression Omnibus repository for differential gene expression (DGE) analysis, comprising human endometrial and placental tissues of patients with recurrent implantation failure (RIF) or recurrent pregnancy loss (RPL). The SCMC genes *KHDC3L*, *NLRP2*, *NLRP4*, *NLRP5*, *OOEP*, *PADI6*, *TLE6*, and *ZBED3* were included in the DGE analysis, as well as *CFL1* and *CFL2* that connect the SCMC with the actin cytoskeleton. Additionally, differential co-expression analysis and systems biology analysis of gene-gene co-expression were performed for *KHDC3L*, *NLRP5*, *OOEP*, and *TLE6*, demonstrating gene pairs differentially correlated under the two conditions, and the co-expression with genes involved in immune response, cell cycle, DNA damage repair, embryo development, and male reproduction. Compared to control groups, *NLRP5* demonstrated upregulation in the endometrium of RIF patients, and *KHDC3L* was upregulated in the fetal placental tissue of RPL patients, shedding light on the importance of considering SCMC genes in reproductive failures.

## Introduction

Infertility is the failure to establish a clinical pregnancy after 12 months of regular and unprotected sexual intercourse, affecting 8-12% of reproductive-aged couples worldwide (Vander Borght and Wyns, 2018). Many factors may lead to infertility, being manifested in different ways, according to the impact on the processes related to human reproduction, whether of maternal, paternal, and/or embryonic origin (Carson and Kallen, 2021). Fertilization failure, embryo arrest, and embryonic implantation failure are some of the reasons for the inability to initiate gestation. However, even once the pregnancy is achieved, its maintenance depends on the correct communication between maternal and embryonic tissues, and abnormalities during this period can lead to pregnancy losses (Ashary et al., 2018). 

Recurrent implantation failure (RIF) is the lack of implantation after the transfer of several embryos through assisted reproductive technologies (Franasiak et al., 2021), whilst recurrent pregnancy loss (RPL) is the failure of two or more clinically recognized pregnancies before 20-24 weeks of gestation (Dimitriadis et al., 2020). However, there is no consensus on the definition of RIF and RPL, varying according to the published guidelines. Both conditions may be related with disturbances in the maternal immune system, genetics of the embryo, anatomic factors, hematologic factors, reproductive tract microbiome, and endocrine environment, as well as endometrial-embryo asynchrony (Dimitriadis *et al.*, 2020; Franasiak *et al.*, 2021). 

The processes involved in early embryo development are regulated and coordinated simultaneously to ensure the generation of a competent embryo capable of sustaining the implantation process and the maintenance of pregnancy (Conti and Franciosi, 2018). During this critical period, specific patterns of gene expression are paramount for regulating cellular proliferation and differentiation, being pivotal for the correct embryo development (Shahbazi and Zernicka-Goetz, 2018). In addition, proper gene expression in the maternal tissues during the time of implantation and pregnancy is also necessary for the changes that this period requires in the maternal reproductive environment (Ashary et al., 2018).

Before the embryo genome activation, the initial development relies almost entirely upon maternal-effect-genes, which have important roles during embryogenesis, such as in the elimination of maternal mRNAs and proteins, epigenetic remodeling in oocytes and early embryos, as well as embryo genome activation (Conti and Franciosi, 2018). Recently, a Subcortical Maternal Complex (SCMC), comprising proteins encoded by maternal effect genes, was identified in mice (Li et al., 2008a) and humans (Zhu et al., 2014), demonstrating fundamental roles in early embryogenesis. Four proteins compose the SCMC: KHDC3L, NLRP5, OOEP, and TLE6. However, the sum of the four proteins (~255 kDa) is smaller than the estimated molecular weight of the SCMC (~669-2000 kDa), hypothesizing that other proteins may be part of the complex, such as the candidates NLRP2, NLRP4F, PADI6, and ZBED3 (Bebbere et al., 2021). In addition, it was demonstrated that the SCMC interacts with the actin cytoskeleton through Cofilin (CFL), regulating symmetric cell divisions of mouse zygotes (Yu et al., 2014). 

The SCMC appears to work as a maternal functional module regulating mammalian early embryogenesis (Lu et al., 2017), however, we hypothesized the individual members of the SCMC could have other roles in human reproduction in addition to embryonic development. Although the SCMC is confirmedly present only in oocytes and early embryos (Li et al., 2008b; Zhu et al., 2014), it is not settled whether the proteins of the SCMC could act as single molecules in other tissues, not being aggregated to form the multi-protein complex. Literature reports based on the evaluation of conditions such as male fertility (Rockenbach et al., 2023), and imprinting disorders (Eggermann et al., 2021), have helped to instigate this hypothesis. However, not limited to tissue variability, we speculate whether the individual members of the SCMC could have roles in processes such as embryonic implantation and even in later steps, such as the maintenance of pregnancy. 

It is well-defined that pregnancy initiation and continuation are regulated by different molecular mechanisms that must be correctly orchestrated between maternal and embryofetal tissues (Ashary et al., 2018). Since the SCMC expression is pivotal for the embryonic genome activation and other initial steps of the pregnancy initiation (Lu et al., 2017), it is coherent to hypothesize that its inactivation might result in an implantation failure (IF). Nevertheless, literature is scarce in regard to the effects of the SCMC in the later gestational period. If the SCMC proteins, acting as a complex or as single molecules, have a role in placentation and endometrial receptivity, it is also feasible to suggest they might be implicated in the recurrent pregnancy loss (RPL) etiology. Therefore, we analyzed the gene expression profile of the SCMC genes, as well as *CFL1* and *CFL2* in endometrial and placental tissues of patients with RIF or RPL through publicly available transcriptomes.

## Material and Methods

### Gene expression analysis

The expression profile of the SCMC genes in RIF or RPL patients was evaluated through differential gene expression (DGE) and differential co-expression analyses of transcriptome data available in the Gene Expression Omnibus (GEO) repository (Edgar et al., 2002; Barrett et al., 2013). For each pathological condition (RIF or RPL), the comparisons were performed against a control group, considering for DGE analysis the gene expression of *KHDC3L, NLRP5, OOEP, TLE6, CFL1, CFL2, NLRP2, NLRP4, PADI6,* and *ZBED3*, and for differential co-expression analysis the expression of *KHDC3L, NLRP5, OOEP,* and *TLE6.*

### Obtention of transcriptome data

For datasets search in the GEO, the keywords “implantation failure”, “pregnancy loss”, “endometrium”, “placenta”, “chorionic villus”, and “decidua” were used, filtering by Entry type (Series), Organism (*Homo sapiens*), and study type (Expression Profile by Array or Expression Profile by Throughput Sequencing). Only studies performed in consolidated platforms, containing the raw data, experimental design, and well-described sample groups were included. 

Following these criteria, three studies were selected, covering endometrium samples of patients with RPL or RIF, as well as placental tissue (chorionic villus or decidua) of RPL patients: GSE26787 (Lédée et al., 2011), GSE121950 (Huang et al., 2018), and GSE113790 (Yu et al., 2018). In the studies selected, the RPL definition was: having at least three pregnancy losses between 6 and 12 weeks of gestations (GSE26787) or two or more consecutive pregnancy losses before 20 weeks of gestations (GSE121950 and GSE113790). RIF was defined as the absence of pregnancy despite the transfer of at least ten embryos over several assisted reproductive cycles (GSE26787). The Control group for endometrial sample of patients with RIF or RPL was fertile patients (successfully delivered after the first or second attempt of IUI or IVF/ICSI related to a male infertility diagnosis). The Control group for chorionic villus or decidua sample of RPL patients consisted of women who underwent legal termination of an apparently normal early pregnancy, without medical reasons, history of pregnancy loss or any pregnancy complication. Additional information about the datasets is available in the supplementary material (Table S1). 

### Differential gene expression analysis

The DGE analysis was conducted in the R environment (v.3.6.3). For the studies comprising RNA-Seq data, sequence alignment was performed through the Galaxy Europe server (Jalili et al., 2020), using the HISAT2 (Kim et al., 2019) alignment tool against the human reference genome hg38 and transcript count was performed through featureCount tool (Liao et al., 2014). The parameters for RNA-Seq data alignment and transcript count were the default ones, and the alignment rate was above 80% for all the samples analyzed. The DGE was calculated in the aligned transcriptomes using the *edgeR* (v.3.28.1) (Robinson et al., 2010) package. Considering microarray data, the packages *affy* (v.1.64.0) (Gautier et al., 2004) and *limma* (v.3.42.2) (Ritchie et al., 2015) were used to evaluate the DGE. RNA-Seq data was normalized through the trimmed mean of M values (TMM) and microarray data by robust multi-array average (RMA). The DGE results are demonstrated as values of log_2_ fold-change (logFC) and adjusted P-value for false discovery rate (FDR), being the DGE considered statistically significant when identified a gene with both log_2_ fold-change (logFC) ≥ |1.0| and adjusted P-value ≤ 0.05. The heatmaps were generated in the R environment through the *ggplot* package (v.3.3.5). 

### Differential co-expression analysis

Additional to the DGE analysis, a differential co-expression analysis was performed considering the basal gene expression of *KHDC3L*, *NLRP5*, *OOEP*, and *TLE6* in control and RIF or RPL patients. Gene-gene co-expression was evaluated using Pearson’s correlation coefficient (Pearson’s r) through the *diffcoexp* package (v.3.17) in the R environment. According to Pearson’s r, a negative correlation coefficient means one gene is upregulated and the other is downregulated; hence, there is an inverse expression between gene pairs. In contrast, positive correlation coefficients mean both genes are upregulated or downregulated. Gene-pairs co-expression was considered moderately correlated when Pearson’s r was ≥ |0.5| and highly correlated when Pearson’s r was ≥ |0.8|. In this study, Pearson’s r was calculated for control samples and then for fertility issues samples (RIF or RPL). The differential correlation between control vs. affected group was calculated through Fisher’s Z transformation method and P-Values < 0.05 was set as significant. Due to the small number of gene-pairs evaluated, no adjustment in the P-Values were performed, but q-Values are presented in Table 1. Hence, gene-pairs were considered differentially co-expressed when there was a significantly different correlation coefficient under the two conditions. As in DGE, the heatmaps for the differential co-expression analyses were generated in the R environment through the *ggplot* package.

**Table 1:** Statistical analysis for the differential co-expression results. Cor (Pearson’s correlation coefficient); diff (differences between control and cases); RPL (recurrent pregnancy loss); RIF (recurrent implantation failure); P-Values ≤ 0.05 were statistically significant.

RECURRENT IMPLANTATION FAILURE VS. CONTROL (ENDOMETRIUM)									
Gene pairs	cor.Control	cor.RIF	cor.diff	p.Control	p.RIF	p.diffcor	q.Control	q.RIF	q.diffcor
*NLRP5 and KHDC3L*	-0.723796175	0.298373579	1.022169754	0.166844543	0.625813633	0.221214199	0.333689086	0.914828318	0.485936692
*OOEP and KHDC3L*	0.524053513	-0.066943717	-0.59099723	0.364696684	0.914828318	0.51636701	0.436785483	0.914828318	0.619640412
*TLE6 and KHDC3L*	0.560272726	0.233327983	-0.326944744	0.325951115	0.705635663	0.692455908	0.436785483	0.914828318	0.692455908
*OOEP and NLRP5*	-0.835869437	0.748757795	1.584627232	0.07782624	0.145340872	0.029448694	0.333689086	0.872045231	0.176692164
*TLE6 and NLRP5*	-0.459018994	0.413211003	0.872229998	0.436785483	0.489264214	0.349507554	0.436785483	0.914828318	0.524261331
*TLE6 and OOEP*	0.753369236	-0.184752233	-0.93812147	0.14146437	0.766111309	0.242968346	0.333689086	0.914828318	0.485936692
RECURRENT PREGNANCY LOSS VS. CONTROL (ENDOMETRIUM)									
Gene pairs	cor.Control	cor.RPL	cor.diff	p.Control	p.RPL	p.diffcor	q.Control	q.RPL	q.diffcor
*NLRP5 and KHDC3L*	-0.768283904	0.152667252	0.920951156	0.129141389	0.806375766	0.242002513	0.468437608	0.909887142	0.889762906
*OOEP and KHDC3L*	0.585454653	-0.070833752	-0.656288404	0.299672502	0.909887142	0.45828449	0.599345004	0.909887142	0.889762906
*TLE6 and KHDC3L*	0.277811088	0.400221583	0.122410495	0.65088408	0.504373014	0.889762906	0.781060896	0.909887142	0.889762906
*OOEP and NLRP5*	-0.73609005	-0.184501747	0.551588303	0.156145869	0.766424755	0.450097613	0.468437608	0.909887142	0.889762906
*TLE6 and NLRP5*	0.098918468	-0.336636046	-0.435554514	0.874258793	0.579620628	0.653044484	0.874258793	0.909887142	0.889762906
*TLE6 and OOEP*	0.385077984	0.533510827	0.148432843	0.522105678	0.354476506	0.85006775	0.781060896	0.909887142	0.889762906
RECURRENT PREGNANCY LOSS VS. CONTROL (CHORIONIC VILLUS)									
Gene pairs	cor.Control	cor.RPL	cor.diff	p.Control	p.RPL	p.diffcor	q.Control	q.RPL	q.diffcor
*NLRP5 and KHDC3L*	0.977945255	-0.913894241	-1.891839495	0.000724254	0.0108021	3.28E-06	0.002172762	0.017799721	9.83E-06
*OOEP and KHDC3L*	0.199428479	0.90968646	0.710257981	0.704823088	0.01186648	0.105011012	0.704823088	0.017799721	0.105011012
*TLE6 and KHDC3L*	-0.942535195	0.972483894	1.915019089	0.004858426	0.001125287	1.82E-06	0.009716851	0.003375862	9.83E-06
*OOEP and NLRP5*	0.389749943	-0.997796689	-1.387546632	0.444977572	7.28E-06	0.000180062	0.533973086	4.37E-05	0.000360123
*TLE6 and NLRP5*	-0.988582995	-0.848216272	0.140366723	0.000194778	0.032809031	0.103260108	0.001168667	0.039370837	0.105011012
*TLE6 and OOEP*	-0.514000802	0.829849741	1.343850543	0.296897486	0.040963646	0.031522489	0.44534623	0.040963646	0.047283734
RECURRENT PREGNANCY LOSS VS. CONTROL (DECIDUA)									
Gene pairs	cor.Control	cor.RPL	cor.diff	p.Control	p.RPL	p.diffcor	q.Control	q.RPL	q.diffcor
*NLRP5 and KHDC3L*	0.060448613	-0.951645102	-1.012093715	0.961493743	0.198783852	1	0.961493743	0.669530442	1
*OOEP and KHDC3L*	0.638595927	-0.836823504	-1.475419431	0.559031102	0.368820066	1	0.746032173	0.669530442	1
*TLE6 and KHDC3L*	-0.860901692	0.764113321	1.625015013	0.339800266	0.446353628	1	0.746032173	0.669530442	1
*OOEP and NLRP5*	0.806737213	0.628175571	-0.178561642	0.402462641	0.567603918	1	0.746032173	0.681124702	1
*TLE6 and NLRP5*	-0.559881268	-0.925333648	-0.36545238	0.621693477	0.247569776	1	0.746032173	0.669530442	1
*TLE6 and OOEP*	-0.941289345	-0.286263143	0.655026202	0.219230836	0.815173694	1	0.746032173	0.815173694	1

### Systems biology analysis

To better elucidate the roles of the SCMC genes in multifactorial conditions such as RIF and RPL, a systems biology approach was conducted for the four validated components of the SCMC: *KHDC3L*, *NLRP5*, *OOEP*, and *TLE6*. A co-expression network was assembled in the Cytoscape (v. 3.8) using the GeneMania application (Montojo et al., 2010), considering only the co-expressed genes filter. 

## Results

**Figure 1 -:**
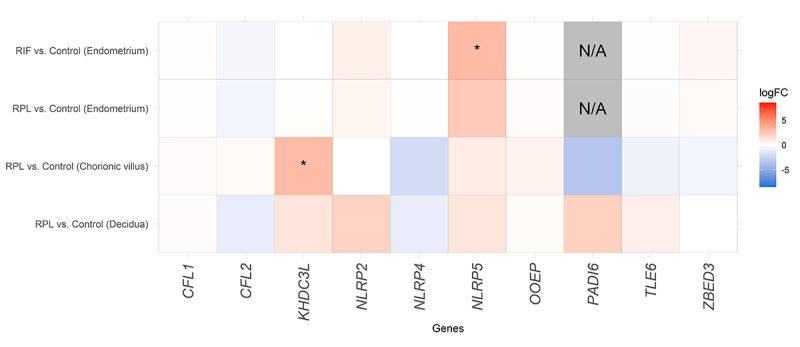
Heatmap of the Subcortical Maternal Complex differential gene expression in endometrial and placental tissues in human reproductive failures. RIF (recurrent implantation failure); RPL (recurrent pregnancy loss); N/A (not available); LogFC (Log_2_ Fold Change); * means statistically significant differences between groups (p ≤ 0.05).

An upregulation of *NLRP5* was observed in the endometrium of patients with RIF compared to the control group (logFC = 3.025; adjusted P-Value = 0.014). Although it was not statistically significant in the other three analyses, *NLRP5* was upregulated in the four scenarios evaluated. Considering the placental tissue, an upregulation of *KHDC3L* was demonstrated in the chorionic villus of RPL patients when compared to the control group (logFC = 3.008; adjusted P-Value = 0.003) (Figure 1). 

Interestingly, no statistically significantly altered genes were observed in the placental samples of maternal origin (decidua) (Adjusted P-Value > 0.05); however, some logFC were increased, demonstrating a differential expression might be present, but without statistical power to confirm it. The values of logFC and adjusted P-Values for all the SCMC genes analyzed are available in Table 2. 

**Table 2:** Statistical analysis for the differential gene expression results. LogFC (log_2_ fold-change); FDR (false-discovery rate); NA (not available); the DGE was considered statistically significant when both logFC ≥ |1.0| and adjusted P-Value ≤ 0.05.

Study	Comparison	Genes	logFC	Adjusted P-value (FDR)
GSE26787	Recurrent implantation failure vs. Control (Endometrium)	*CFL1*	0.1026367	0.5229698
		*CFL2*	-0.498079897867953	0.251416316493106
		*KHDC3L*	-0.001182168	0.9962905
		*NLRP2*	0.681588601771906	0.681588755422925
		*NLRP4*	0.009650816	0.9714375
		***NLRP5***	**3.02522473023772**	**0.0141471070182118**
		*OOEP*	0.111747	0.8106413
		*PADI6*	NA	NA
		*TLE6*	0.02474961	0.9497394
		*ZBED3*	0.379691270618091	0.559698817131491
	Recurrent pregnancy loss vs. Control (Endometrium)	*CFL1*	0.0786863159624627	0.59939475537244
		*CFL2*	-0.582603971710068	0.277270727132137
		*KHDC3L*	0.0811707017617804	0.75759904538884
		*NLRP2*	0.457898306429951	0.696673633291216
		*NLRP4*	0.0259633183126278	0.918007451161419
		*NLRP5*	2.34493710842825	0.251792093808216
		*OOEP*	0.117712311568029	0.787288812071011
		*PADI6*	NA	NA
		*TLE6*	-0.148942627713211	0.485773545319927
		*ZBED3*	0.259967817024501	0.549032896179686
GSE121950	Recurrent pregnancy loss vs. Control (Chorionic villus)	*CFL1*	0.207979556312718	0.39350378872339
		*CFL2*	0.211804830319393	0.704923759930941
		***KHDC3L***	**3.00853375453727**	**0.00293672701411503**
		*NLRP2*	-0.00893939223730675	1
		*NLRP4*	-2.02451760996167	0.370388208966524
		*NLRP5*	0.858074625612382	0.575326063792963
		*OOEP*	0.646653028452488	0.542039977168646
		*PADI6*	-3.35312117601586	0.160290137066962
		*TLE6*	-0.71115056497841	0.249818659395077
		*ZBED3*	-0.541428483402332	0.605711244872257
GSE113790	Recurrent pregnancy loss vs. Control (Decidua)	*CFL1*	0.122166824419392	1
		*CFL2*	-1.03283267355495	0.442221956087787
		*KHDC3L*	1.09732315067292	0.954460730152779
		*NLRP2*	1.97492180673471	0.831821230943147
		*NLRP4*	-0.999040419492552	0.986849232233079
		*NLRP5*	1.08159364196709	0.789621847412384
		*OOEP*	0.182337399040928	1
		*PADI6*	2.03571376974121	0.831795991373028
		*TLE6*	0.69944683541296	0.815358493093605
		*ZBED3*	0.00907890935220243	1

**Figure 2 -:**
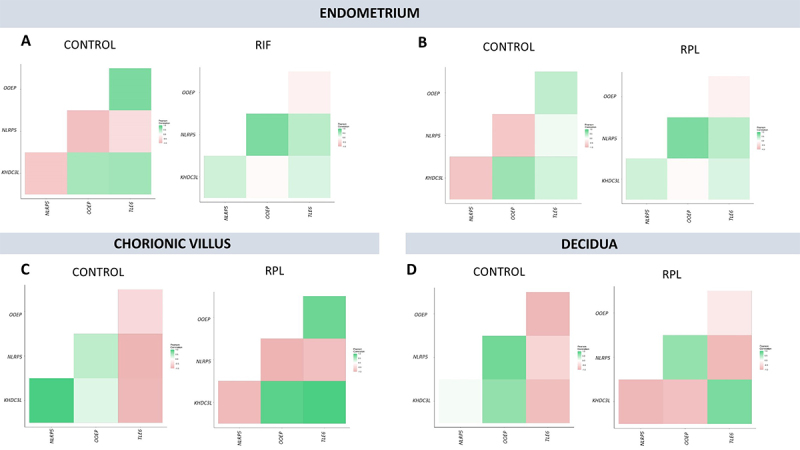
Differential co-expression analysis for the four validated Subcortical Maternal Complex genes in the endometrium of control and RIF patients (A), endometrium of control and RPL patients (B), chorionic villus of control and RPL patients (C), and decidua of control and RPL patients (D). Positive correlations represented in green; negative correlations in pink; absence of correlation in white. RIF (recurrent implantation failure); RPL (recurrent pregnancy loss).

Pearson’s r was calculated to evaluate the co-expression between gene-pairs, considering the four genes of the SCMC complex. It was observed that the gene expression correlation between *KHDC3L*, *NLRP5*, *OOEP*, and *TLE6* is lost in the endometrium of RIF patients compared to the control group (Figure 2A), although it was not statistically different when applying Fisher’s-Z transformation. Except for *TLE6* and *NLRP5* (Pearson’s r < 0.5), all the other gene-pairs demonstrated a moderate or high correlation in the control group. However, in RIF patients these correlations were lost, except for *OOEP* and *NLRP5,* which significantly inverted the correlation pattern, from a high inverse correlation to a moderate positive correlation (Control = -0.84 vs. RIF = 0.75, P-Value = 0.03). Considering the endometrium of RPL patients, no statistically significant differences in SCMC genes’ co-expression were observed (Figure 2B). 

Comparing the chorionic villus of RPL and control patients, it was observed a statistically significant different correlation between *NLRP5* and *KHDC3L* (Control = 0.98 vs. RPL = -0.91, P-Value = 3,28E-06), *TLE6* and *KHDC3L* (Control = -0.94 vs. RPL = 0.97, P-Value = 1.82E-06), *OOEP* and *NLRP5* (Control = 0.39 vs. RPL = -0.99, P-Value = 0.0002), and *TLE6* and *OOEP* (Control = -0.51 vs. RPL = 0.83, Adjusted P-Value = 0.03) (Figure 2C). Interestingly, in the placental maternal tissue (decidua) it was not observed statistically significant differences in gene expression correlation between RPL and control patients (Figure 2D). The statistical analysis results for differential co-expression analysis are available in Table 1. 

**Figure 3 -:**
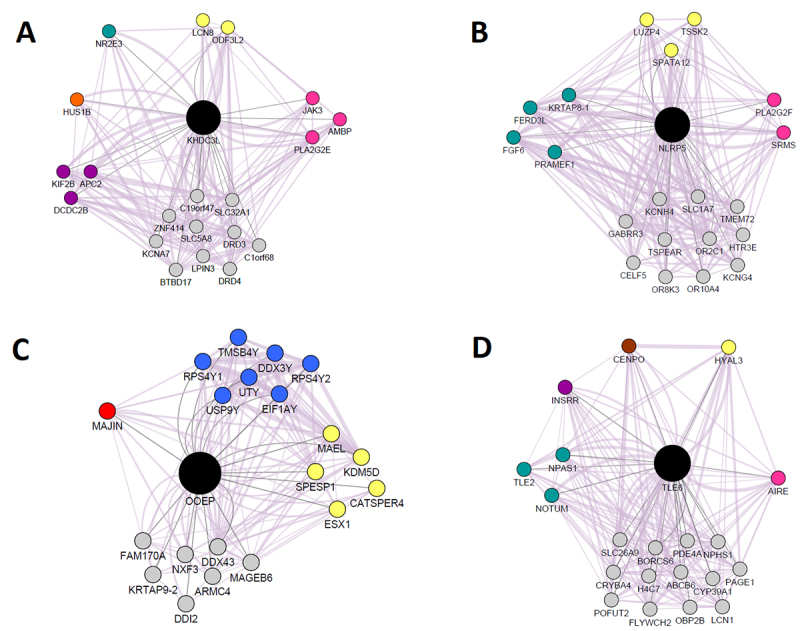
Co-expression network for the four validated Subcortical Maternal Complex genes. A) *KHDC3L* co-expression network; B) *NLRP5* co-expression network; C) *OOEP* co-expression network; D) *TLE6* co-expression network. Nodes of different colors means distinct biological functions and edges connecting nodes represent genes that are co-expressed. Pink: genes related to immune response; yellow: male reproduction; green: embryonic development; purple: cytoskeleton organization; orange: DNA damage response; brown: cell cycle and chromosome segregation; red: meiosis; blue: located in Y chromosome; grey: other functions.

Gene-gene co-expression analysis for *KHDC3L*, *NLRP5*, *OOEP*, and *TLE6* was also evaluated through systems biology strategy. It was demonstrated the SCMC genes interact with genes related to DNA damage response and repair, embryo development, immune response, cell division, chromosome segregation, and male reproduction (Figure 3). The four SCMC genes were co-expressed with genes related to male reproduction, especially *OOEP* which was also co-expressed with genes located in the Y chromosome. Additionally, except for *OOEP*, all the other genes were co-expressed with genes associated with immune response, and only *KHDC3L* was co-expressed with a gene related to DNA damage repair. 

## Discussion

Embryo implantation and maintenance of pregnancy depend on a competent blastocyst, receptive endometrium, and successful cross-talk between the embryonic and maternal interfaces (Ashary et al., 2018). Here we demonstrated, through publicly available data, altered SCMC gene expression and co-expression patterns in RIF and RPL patients. Compared to the control groups, an upregulation of *NLRP5* was demonstrated in the endometrium of patients with RIF, as well as an upregulation of *KHDC3L* in the chorionic villus of RPL patients. Additionally, we demonstrated that *KHDC3L*, *NLRP5*, *OOEP*, and *TLE6* are being co-expressed with genes involved in processes required for proper embryo development and gestational maintenance, such as immune response, cell proliferation, and DNA damage repair. 

The SCMC exerts several functions during early embryo development, being required for embryo progression beyond the first cell divisions (Li et al., 2008a; Zhu et al., 2014). However, studies have demonstrated alterations in SCMC genes associated with later reproductive problems, such as recurrent hydatidiform mole (Ji et al., 2019), RPL (Zhang et al., 2019), and multilocus imprinting disorders (Docherty et al., 2015). Although the molecular mechanisms behind reproductive disorders and SCMC genes remain poorly understood, it is feasible to suggest that SCMC gene expression is important not only during early embryogenesis but also later in pregnancy. 

During the implantation process, the receptive endometrium is modified by embryonic signals accompanied by substantial morphological, molecular, and immunological changes required for proper embryo implantation and further maintenance of pregnancy (Ashary et al., 2018). We demonstrated an upregulation of *NLRP5*, a member of the SCMC, in the endometrium of RIF patients in comparison to the control group, as well as *NLRP5* co-expression with genes related to immunological processes. Early pregnancy modulates the expression of the NLR family in ovine lymph nodes (Zhao et al., 2022), evidencing a role for this protein family in maternal immune regulation during pregnancy. Considering NLR family of proteins have a role in the activation of pro-inflammatory cytokines (Platnich and Muruve, 2019) and embryo implantation is considered a pro-inflammatory reaction characterized by increased endometrial vascular permeability and trophoblast invasion (Kim and Kim, 2017), we postulate *NLRP5* upregulation in endometrial cells may affect embryo implantation through altered immunological regulation. 

Gene variants in *NLRP5*, as well as in other genes of the SCMC, are associated with embryo arrest (Mu et al., 2019; Xu et al., 2020). Additionally to this phenotype, alterations in *NLRP5* have been associated with multilocus imprinting disorders in humans, a disturbance of multiple imprinting locus across the genome affecting metabolism, growth, and behavior (Docherty et al., 2015; Sparago et al., 2019). Epigenetic regulation of gene expression has a role in embryo implantation and gestational maintenance by regulating both embryo development and endometrial changes required for successful implantation (Munro et al., 2010; Xu *et al.*, 2021). Although the mechanisms behind methylation defects associated with mutations in *NLRP5* remain to be elucidated, this gene could be involved in the epigenetic regulation of endometrial gene expression during embryo implantation. Therefore, we hypothesized that upregulation of *NLRP5* could be associated with epigenetic deregulation of genes important during the pro-inflammatory scenario necessary for the trophoblast invasion and embryo implantation, thereby affecting the activation of pro-inflammatory cytokines. 

However, after embryo implantation and establishment of pregnancy, its maintenance depends not only on proper embryo development but also on the correct maternal-embryo communication (Ashary et al., 2018). In this context, the correct formation of the placenta, an extraembryonic organ crucial for normal development and long-term health, is pivotal for gestational maintenance (Knöfler et al., 2019). Around 5-6 days after fertilization, the blastocyst develops and segregates into two cellular subtypes: the trophectoderm, which will differentiate to form the embryonic placental tissue - chorionic villus -, and the inner cell mass giving rise to the embryo proper (Knöfler *et al.*, 2019). After blastocyst implantation, placental development is initiated and trophectoderm-derived cells give rise to all trophoblast cell types of the future placenta (Woods et al., 2018). In addition to the fetal-derived cells, the placental development is also dependent on the maternal uterine tissue into which the blastocyst is embedded after implantation (Woods *et al.*, 2018). The cells of the endometrium undergo decidualization, which is pivotal for supporting normal placentation and providing the proper environment for embryonic growth and survival (Woods *et al.*, 2018). Interestingly, we demonstrated an upregulation of *KHDC3L* in the chorionic villus of patients with RPL in comparison to the control group. Although *KHDC3L* mRNAs are rarely detected in human morulae, the transcript’s level increases dramatically in the blastocyst, and like the other members of the SCMC, its location in the blastocyst stage is exclusive of the outer layer formed by the trophectoderm (Li et al., 2008b; Zhu et al., 2014). The specific localization of the SCMC during early embryo development could be associated with a role in lineage cell decisions during development and, in this context, *KHDC3L* could be related to the trophoblast cells proliferation and differentiation involved in placental development. 

In addition, variants in *KHDC3L* have been associated with hydatidiform moles, an abnormal pregnancy characterized by abnormal trophectoderm proliferation and abnormal or no embryo development (Ji et al., 2019; Demond et al., 2019). This evidence demonstrates a possible role of *KHDC3L* in the trophectoderm proliferation and differentiation, which further could affect the placental development. Indeed, failures in placental formation can compromise embryonic growth and development, and abnormal placentation is a feature of diverse pregnancy complications such as pregnancy loss, stillbirth, intrauterine growth restriction, and preeclampsia (Knöfler et al., 2019). *KHDC3L* variants have also been related to imprinting disturbance and genomic instability of early embryonic cells leading to reproductive failures, including RPL (Zhang et al., 2019). Indeed, *KHDC3L* has a role in safeguarding genome integrity through homologous DNA repair (Zhang *et al.*, 2019) and stalled replication fork restart (Zhao et al., 2018). Therefore, we hypothesized that upregulation of *KHDC3L* in chorionic villus could be associated with altered epigenetic regulation of genes related to DNA repair mechanisms, thereby disturbing trophoblast cell proliferation and differentiation, influencing the proper placental development.

Interestingly, considering *KHDC3L*, *NLRP5*, *OOEP*, and *TLE6*, it was observed that except for *TLE6* and *NLRP5*, all the other gene-pairs demonstrated a moderate or high correlation in the endometrium of control group. However, in RIF patients these correlations were lost. Furthermore, *OOEP* and *NLRP5* significantly inverted the correlation pattern, from a high negative correlation to a moderate positive correlation, which could be related to the upregulation of *NLRP5* demonstrated in the DGE results. Alterations in gene expression correlation were also observed between the gene-pairs *NLRP5* and *KHDC3L*, *TLE6* and *KHDC3L*, *OOEP* and *NLRP5*, and *TLE6* and *OOEP* in the chorionic villus of RPL patients. Considering the role of gene expression patterns during embryo development, the disrupted gene-gene co-expression demonstrated in RIF and RPL patients could influence the proper embryo implantation and gestational maintenance. Although we cannot confirm a causal association between RIF and RPL with altered co-expression patterns in the four validated SCMC genes, a transcriptional deregulation of these genes is present in these conditions and even if it is not associated with RIF and RPL, tertiary factors could be influencing this deregulation. 

Additional to the differential co-expression analysis performed for *KHDC3L*, *NLRP5*, *OOEP*, and *TLE6*, we evaluated the co-expression of the SCMC members with other genes. Interestingly, the co-expression network demonstrated that the four validated SCMC genes are co-expressed with genes related to male reproduction. Interestingly, a previous work of our group evidenced *OOEP* downregulation in patients with teratozoospermia or non-obstructive azoospermia (Rockenbach et al., 2023), evidencing possible new roles for the SCMC genes in both embryonic development and in female and male reproduction. Moreover, the results presented here shed light on a possible role of SCMC gene expression profile in later reproductive conditions, such as in post-implantation gestational events.

This study has some limitations, such as the lack of validation of the data analyzed and the absence of single-cell transcriptome studies. Functional analysis needs to be performed to demonstrate the mechanisms behind the gene expression alterations of *NLRP5* and *KHDC3L* in RIF and RPL patients, respectively, as well as in the altered co-expression patterns observed for these conditions. It is also important to highlight the biases of clinical differences between the datasets used in this study, such as the different definitions for RPL. However, the results presented here shed light on possible molecular mechanisms associated with reproductive failures and demonstrate the importance of considering the roles of the SCMC genes in different scenarios, as well as the role of gene expression profiles in the beginning of pregnancy. Besides, the co-expression network performed for *KHDC3L*, *NLRP5*, *OOEP*, and *TLE6* demonstrated their co-expression with genes related to different biological processes involved in human reproduction, such as DNA damage response and repair, embryo development, immune response, cell division, chromosome segregation, and male reproduction. Therefore, although the SCMC is confirmedly present in oocytes and early embryos, the components of this complex may exert different reproductive roles in different scenarios and may be considered in future studies aiming to understand reproductive failures of both embryonic, maternal and/or paternal origin. 

## Supplementary material

The following online material is available for this article:

Table S1 - Datasets selected for the differential gene expression analysis.
