# Supplementary material for: Revealing the expression profile of genes that encode the Subcortical Maternal Complex in human reproductive failures
Source: Genet Mol Biol. 2023 Dec 11;46(3 Suppl 1):e20230141. doi: 10.1590/1678-4685-GMB-2023-0141 (PMC10718294; doi:10.1590/1678-4685-GMB-2023-0141)
Supplement: Table S1 - [file 1415-4757-GMB-46-03-s1-e20230141-s1.pdf]

Genetics and Molecular Biology
https://doi.org/10.1590/1678-4685-GMB-2023-0141

Supplementary Material to "Revealing the expression profile of genes that encode the Subcortical Maternal Complex in human reproductive failures"

Table S1 - Datasets selected for the differential gene expression analysis.

Assay	Comparison	Tissue	Cases definition	Control group	Technology	N^a	N^b	PMID
GSE26787	Recurrent implantation failure[a] vs. Control[b]	Endometrium	RIF: absence of pregnancy after the transfer of at least ten embryos over several assisted reproductive cycles	Fertile patients	Microarray	4	5	22025212
	Recurrent pregnancy loss[a] vs. Control[b]		RPL: at least three pregnancy losses between 6 and 12 weeks of gestations			4	5	
GSE121950	Recurrent pregnancy loss[a] vs. Control[b]	Chorionic villus	RPL: two or more consecutive pregnancy losses before 20 weeks of gestations	Legal termination of an apparently normal early pregnancy	RNASeq	6	6	30448228
GSE113790	Recurrent pregnancy loss[a] vs. Control[b]	Decidua	RPL: two or more consecutive pregnancy losses before 20 weeks of gestations	Legal termination of an apparently normal early pregnancy	RNASeq	3	3	30100398
